# Tumor mutational load, CD8^+^ T cells, expression of PD-L1 and HLA class I to guide immunotherapy decisions in NSCLC patients

**DOI:** 10.1007/s00262-020-02506-x

**Published:** 2020-02-12

**Authors:** Daan P. Hurkmans, Merian E. Kuipers, Jasper Smit, Ronald van Marion, Ron H. J. Mathijssen, Piet E. Postmus, Pieter S. Hiemstra, Joachim G. J. V. Aerts, Jan H. von der Thüsen, Sjoerd H. van der Burg

**Affiliations:** 1 Department of Pulmonary Medicine, Erasmus University Medical Center, Doctor Molewaterplein 40, 3015 GD Rotterdam, The Netherlands; 2 Department of Medical Oncology, Erasmus University Medical Center, Erasmus MC Cancer Institute, Rotterdam, The Netherlands; 3 Department of Pulmonology, Leiden University Medical Center, Leiden, The Netherlands; 4 Department of Pathology, Erasmus University Medical Center, Rotterdam, The Netherlands; 5 Department of Medical Oncology, Leiden University Medical Center, Leiden, The Netherlands

**Keywords:** Nivolumab, NSCLC, TMB, Tumor microenvironment, Biomarker

## Abstract

**Objectives:**

A minority of NSCLC patients benefit from anti-PD1 immune checkpoint inhibitors. A rational combination of biomarkers is needed. The objective was to determine the predictive value of tumor mutational load (TML), CD8^+^ T cell infiltration, HLA class-I and PD-L1 expression in the tumor.

**Materials and methods:**

Metastatic NSCLC patients were prospectively included in an immune-monitoring trial (NTR7015) between April 2016-August 2017, retrospectively analyzed in FFPE tissue for TML (NGS: 409 cancer-related-genes) and by IHC staining to score PD-L1, CD8^+^ T cell infiltration, HLA class-I. PFS (RECISTv1.1) and OS were analyzed by Kaplan–Meier methodology.

**Results:**

30 patients with adenocarcinoma (67%) or squamous cell carcinoma (33%) were included. High TML was associated with better PFS (*p* = 0.004) and OS (*p* = 0.025). Interaction analyses revealed that patients with both high TML and high total CD8^+^ T cell infiltrate (*p* = 0.023) or no loss of HLA class-I (*p* = 0.026), patients with high total CD8^+^ T cell infiltrate and no loss of HLA class-I (*p* = 0.041) or patients with both high PD-L1 and high TML (*p* = 0.003) or no loss of HLA class-I (*p* = 0.032) were significantly associated with better PFS. Unsupervised cluster analysis based on these markers revealed three sub-clusters, of which cluster-1A was overrepresented by patients with progressive disease (15 out of 16), with significant effect on PFS (*p* = 0.007).

**Conclusion:**

This proof-of-concept study suggests that a combination of PD-L1 expression, TML, CD8^+^ T cell infiltration and HLA class-I functions as a better predictive biomarker for response to anti-PD-1 immunotherapy. Consequently, refinement of this set of biomarkers and validation in a larger set of patients is warranted.

**Electronic supplementary material:**

The online version of this article (10.1007/s00262-020-02506-x) contains supplementary material, which is available to authorized users.

## Introduction

Tumors evade T-cell mediated destruction by exploiting inhibitory immune checkpoints such as the PD-1/ PD-L1 pathway. The efficacy of treatment with immune checkpoint inhibitors (ICIs) targeting this pathway in non–small-cell lung cancer (NSCLC) is limited, and better use of biomarkers is needed to predict response to treatment [3].

The currently most widely used biomarker is PD-L1 expression in the tumor, as assessed by the PD-L1 tumor proportion score (TPS), which is positively associated with a response to ICI treatment in metastatic NSCLC patients [4]. However, the performance of the PD-L1 assay to predict clinical response remains poor [5].

The presence of tumor-infiltrating CD8^+^ T cells which recognize tumor antigens, when presented at the tumor cell surface in the context of HLA class I, is a prerequisite for successful ICI treatment. A surrogate marker for recognition of tumor antigens is tumor mutational load (TML), a measurement of the frequency of mutations in tumor cells, that correlates with the number of neoantigens that can be recognized by CD8^+^ T cells [6]. Next-generation genome sequencing (NGS) panels composed of about 300–600 cancer-related genes are designed to predict the TML with similar accuracy as whole-exome sequencing [7]. A strong CD8^+^ type 1 T cell infiltration of tumors critically contributes to a better clinical outcome in cancer, including NSCLC [6]. Conversely, (partial) loss of HLA occurs in a sizeable fraction of NSCLC tumors, as well as HLA diversity modulate the prognostic impact of tumor-infiltrating CD8^+^ T cells, and thus survival after checkpoint blockade [8–11]. While TML is an emerging biomarker, CD8^+^ T cell infiltration and HLA expression have not been considered as predictive biomarkers in NSCLC.

Therefore, this study is the first to determine the predictive value of the TML, CD8^+^ T cells and HLA class I expression in combination with the PD-L1 expression in anti-PD-1 treated NSCLC.

## Materials and methods

### Study population

Patients with stage IV NSCLC who started nivolumab monotherapy between April 2016 and August 2017 at the Erasmus University Medical Center, Rotterdam, The Netherlands, were included prospectively in the MULTOMAB study (Dutch Trial Registry NTR7015/NL6828). The study was approved by the independent ethics committee (Medical Research Ethics Committee Erasmus MC; MEC 16-011) and all patients provided written informed consent. Patients were randomly selected and assessed for eligibility. Patients with NSCLC stage IV were included who had been treated with nivolumab monotherapy (weight-based dosing: 3 mg/kg i.v., Q2W) and who were evaluable by RECIST v1.1. Patients who were treated with a prior line of immunotherapy were excluded. The median follow-up time was 27 weeks (interquartile range 14–46 weeks) and the median time between the diagnostic biopsy and first administration of nivolumab was 5 weeks (interquartile range 1–41 weeks). The use of archival formalin-fixed, paraffin-embedded (FFPE) samples was in accordance with guidelines from the Dutch Federation of Medical Research and was approved by the independent ethics committee (Medical Research Ethics Committee Erasmus MC; MEC 17-1186). Specimen handling and all biomarker assay analyses were undertaken blinded; a unique code was assigned for each patient, with a separate list linking these codes with the patient characteristics and outcomes.

### TML assay

Mutational load was determined by the Oncomine TML assay (ThermoFisher Scientific, Waltham, MA) according to manufacturer’s protocol on an Ion Torrent S5 XL next-generation sequencing platform (Gilford, NH). Mutational load is defined as the number of somatic nonsynonymous variants (missense and nonsense single nucleotide variants plus insertions and deletions) detected per megabase of exonic sequence with sufficient coverage. Germline variants were filtered out using the Mutation Load Calculation Filter Chain in Ion Reporter software 5.10 (ThermoFisher Scientific, Waltham, MA). A TML cut-off of 11 mut/Mb was used to differentiate between tumors with low or high TML.

### Immunohistochemistry on FFPE samples

Expression patterns of classical HLA (HLA-A and HLA-B/C) were assessed according to the Ruiter scoring system [12] as described before [10]. The intensity and percentage of cells in the tumor were determined based on the sum of the intensity of staining (ranging from 0–3) and percentage positive cells (ranging from 0–5). Loss of HLA class I was defined by a low Ruiter score (0–3) of both HLA-A and HLA-B/C. Patients were dichotomized for low or high total CD8^+^ T cell infiltration based on the mean CD8^+^ T cell infiltration for all patients and for low (< 50%) or high (≥ 50%) PD-L1 TPS (using the ready-to-use SP263 Ab clone on a Ventana Benchmark Ultra (both form Roche Diagnostics, Tucson, AZ) system according to the manufacturer’s instructions. Mouse monoclonal Abs HCA-2 and HC-10 (tissue culture supernatant respectively anti HLA-A, 1:500, and anti HLA-B/-C, 1:750; a generous gift from Prof. dr. J. Neefjes, Department of Cell and Chemical Biology, LUMC) were used to detect the free heavy chain of the classical HLA-A and HLA- B/-C molecule). The detection of CD8^+^ T-cells was done using mouse monoclonal CD8 Ab (clone IA5, Leica Biosystems, Germany, 1:500). PD-L1 TPS was determined using clone SP263 (Ventana PD-L1 assay, Roche, Switzerland)**.**

### Statistical analysis

Best overall response (BOR) was assessed according to RECIST v1.1 for complete response (CR), partial response (PR), stable disease (SD) and progressive disease (PD): minimum duration of 90 days for SD was required, confirmation of CR or PR was not necessary. PFS was defined as the time between the first administration of nivolumab until PD or death due to any cause, and OS until death due to any case. Survival was compared by log-rank test using Kaplan–Meier methodology. Group comparisons of categorical data were performed by 2-tailed *χ*^2^ or Fisher’s Exact test. Differences with two-sided *P* values < 0.05 were considered significant. No power analysis was performed in this proof-of-concept study. *R* version 1.1.453 (*R*-project, www.rproject.org) was used for hierarchical cluster analysis with complete linkage by Manhattan distance measure, using the mean for missing values, statistical software package SPSS v24.0.0.1 (SPSS, Chicago, USA) was used for further statistical analysis.

## Results

**Fig. 1:**
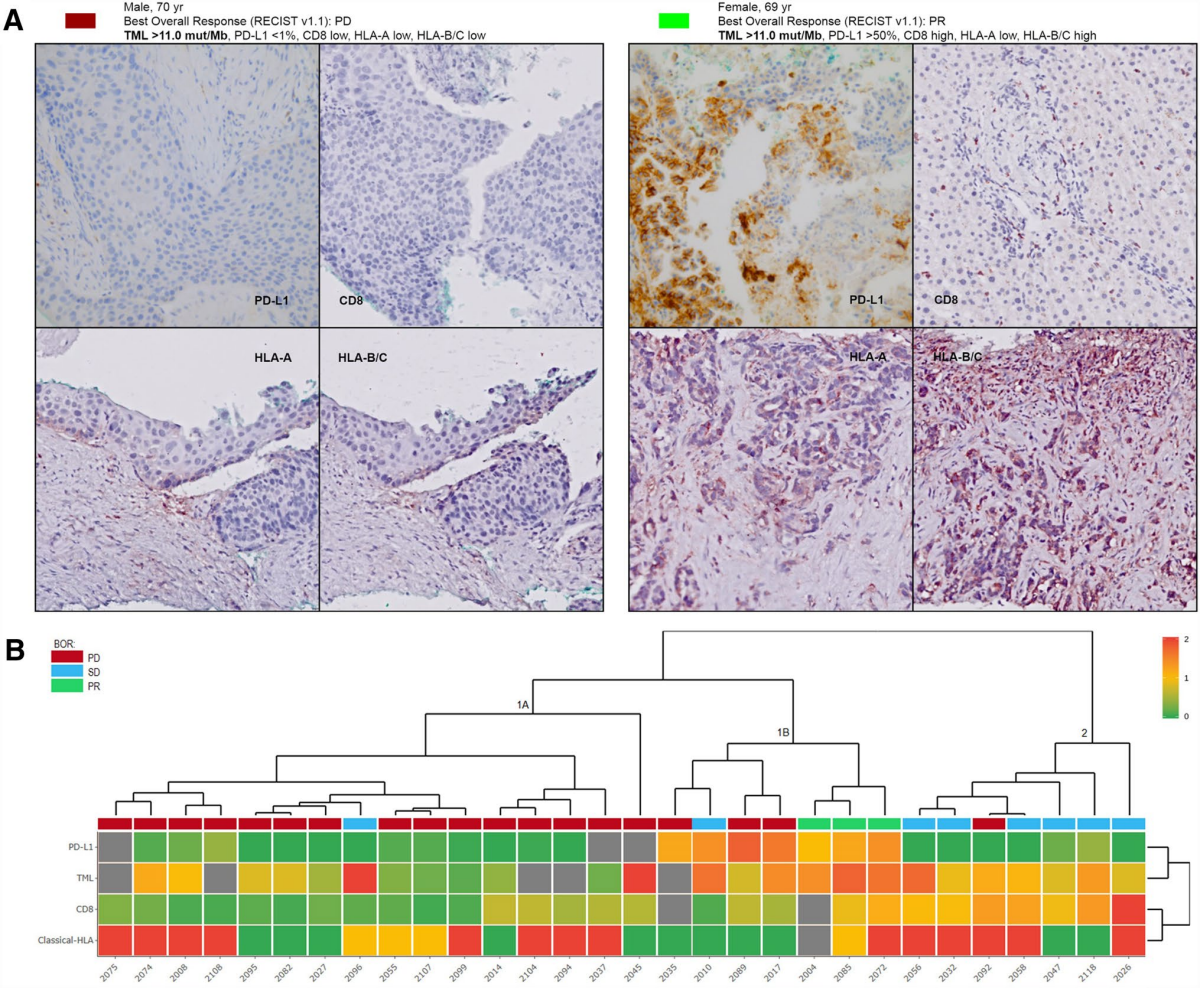
Patient examples and cluster analysis. a Example of two patients showing the BOR (RECIST v1.1): TML NGS output, and the IHC of HLA-A, HLA-B/C, total CD8^+^ and PD-L1. For HLA, the percentage of positive tumor cells was classified (0–5) and the intensity of the staining (0–3), resulting in a final score based on both (0–8) and was categorized as 0–3 (low) or 4–8 (high); according to the Ruiter scoring system. Loss of classical HLA was defined as absent expression (0–3) of both HLA-A and HLA-B/C IHC. Magnification × 20. **b** Heat map of unsupervised cluster analysis based on classical HLA, total CD8 tumor infiltration, TML and PD-L1 revealing three distinct clusters (1–3). BOR by RECIST v1.1 was incorporated in the heat map

A total of 99 patients were assessed for eligibility, of whom 69 patients were excluded because of either insufficient or poor quality of samples (*n* = 33), or failure to obtain FFPE material from referring hospitals (*n* = 36). 30 patients were analyzed (Table 1). The mean duration of nivolumab treatment was 5.4 months (SD: 4.6). Two patients (6%) developed severe immune-related toxicity (grade 3/4, according to CTCAE 4.03). All patients had at least one prior line of chemotherapy, consisting of platinum-based doublet chemotherapy, and three patients were also treated with an EGFR tyrosine kinase inhibitor. The mean duration of response to first-line chemotherapy was 7.2 months (SD: 4.7). Examples of two representative patients are shown in Fig. 1a, displaying TML and IHC staining of classical HLA, CD8^+^ T cells and PD-L1.

**Table 1:** Patient characteristics, TML and IHC patterns at baseline

Patient characteristics	Number	Mean (SD)
Age (years)	30	64(8.6)

	Number	(%)
Gender		
Male	18	(60.0)
Female	12	(40.0)
NSCLC type		
SCC	10	(33.3)
Adenocarcinoma	20	(66.6)
Regimen		
Nivolumab	30	(100)
Prior chemotherapy		
Yes	30	(100)
History of smoking		
Yes	23	(88.5)
No	2	(11.5)
TML		
Low	17	(68.0)
High	8	(32.0
HLA-A		
Low	14	(48.3)
High	15	(51.7)
HLA-B/C		
Low	12	(41.4)
High	17	(58.6)
Total CD8^+^		
Low	16	(57.1)
High	12	(42.9)
PD-L1 (TPS)		
Neg (0%)	11	(40.7)
Pos (≥ 1%)	16	(59.3)

From a total of 99 eligible patients, 69 were non-evaluable for this analysis, because either there was no sufficient archived FFPE tissue (*n* = 31), FFPE tissue could not be obtained from the referring hospital (*n* = 36) or the tissue was of poor quality (*n* = 2). The expression patterns of HLA-A and HLA-B/C as well as the total CD8^+^ T cell infiltration in these patients were similar to what we reported before in a comparable group of NSCLC patients [10]

**Fig. 2:**
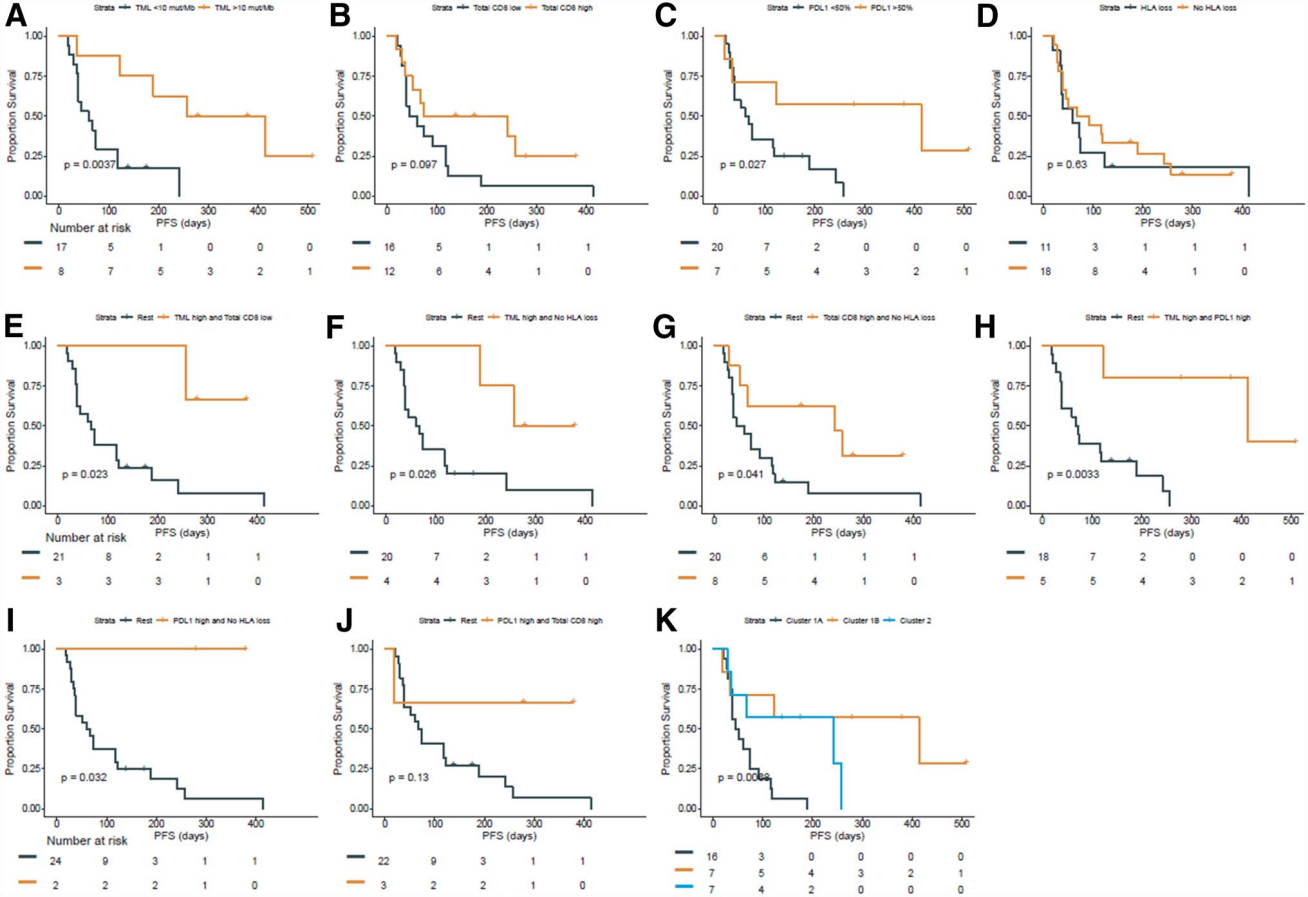
PFS analysis. Kaplan–Meier plots showing the PFS by **a** TML high (> 11 mut/Mb) vs. low (< 11 mut/Mb), **b** CD8^+^ T cell infiltration high vs. low, **c** PD-L1 high (> 50%) vs low, **d** classical HLA (-A and –B/C) loss vs. rest, **e** TML high and total CD8^+^ high vs. rest, **f** TML high and no loss of classical HLA vs. rest, **g** total CD8^+^ high and no loss of classical HLA vs. rest, **h** TML high and PD-L1 high vs. rest, **i** PD-L1 high and no loss of classical HLA vs. rest, **j** PD-L1 high and CD8^+^ high vs. rest and **k** cluster (cluster 1A, 1B and 2)

First, the prognostic effect of each parameter on PFS (Fig. 2a–d) and OS (Fig. S1a-d) was determined. High TML was significantly associated with better PFS (*p* = 0.004) and OS (*p* = 0.025). PD-L1 was associated with improved PFS (*p* = 0.027), but not with OS (*p* = 0.121). CD8^+^ T cells and HLA as individual biomarkers were not significantly associated with better OS or PFS, which was expected [10], although normal expression of HLA class I resulted in the better OS and PFS when compared to complete or partial loss of HLA expression. Complete loss was defined by a low score (0–2), partial loss by an intermediate score (3–6), and normal expression by a high score (7–8). Patients with complete loss had impaired PFS compared to patients with partial loss or normal expression of HLA class I (Fig. S2).

However, interaction analyses between these two markers and the other markers revealed that each combination of two markers was significantly associated with better PFS (Fig. 2e–j), except for PD-L1 with CD8^+^ T cells due to low power. Specifically, (1) high TML and either high total CD8^+^ T cell infiltration (*p* = 0.023) or no loss of HLA class I (*p* = 0.026), (2) high total CD8^+^ T cell infiltration and no loss of HLA class I (*p* = 0.041), and (3) high PD-L1 and either high TML (*p* = 0.003) or no loss of HLA class I (*p* = 0.032) was associated with better PFS.

Next, the patients were divided into two groups on the basis of a clinical response (CR/PR/SD) or failure to respond (PD) to nivolumab treatment (Table S1). This revealed a significant overrepresentation of patients with a high TML (*p* = 0.043) and/or more profound total CD8^+^ T cell infiltration (*p* = 0.005) among clinical responders. This association was not found for HLA class I expression or PD-L1 (TPS ≥ 1% or ≥ 50%).

A comparison of the absolute values for all these parameters confirmed that the mean TML (*p* = 0.001) and mean total CD8^+^ T cell infiltrate (*p* = 0.004) were higher in the group of patients with a treatment response (Table S2). Notably, the TML was not directly correlated with CD8^+^ T-cell infiltration or HLA expression but was positively associated with PD-L1 (*p* = 0.035; Table S3).

Finally, an unsupervised cluster analysis based on the four parameters was performed. This revealed two major clusters and a total of three sub-clusters (Fig. 1b). Cluster 1A was overrepresented by patients with PD (15 out of 16) of which the tumors were negative for 2–3 of the 4 biomarkers. Cluster 2 almost exclusively comprised patients with SD of which the tumor was positive for 2–3 biomarkers but did not express PD-L1. Survival analyses of the 3 different clusters (Figs. 2k, S1k) indicate a significant effect on PFS (*p* = 0.007), while the OS (*p* = 0.74) was significant in a post-hoc comparison of cluster 1A with 1B (*p* = 0.048).

## Discussion

The present proof-of-concept study suggests that in addition to PD-L1 expression also TML, CD8^+^ T cell infiltration and HLA class I expression are associated with PFS and predict the response to anti-PD-1 immunotherapy. Interestingly, unsupervised cluster analysis of the patients based on all four markers revealed one cluster pattern that almost exclusively identified non-responders (cluster 1A). In the current real-life setting, only a small amount of archival material could be used for TML determination and IHC staining, derived from routine biopsy specimens from the primary tumor and following initial diagnostic procedures (including routine NGS testing for driver mutations in some cases). We were able to demonstrate the clinical value of TML analysis in this immuno-oncology setting, and we believe this is the first study to do so in combination with a range of IHC biomarkers in small, realistic biopsy specimens.

Our findings are consistent with previous studies in different settings, although those studies cannot be generalized. Associations between TMB and immune signatures are generally cancer type dependent [13], and it can be assumed that they are also tumor stage dependent. In addition, a prospective study in early-stage untreated NSCLC patients demonstrated a significant and independent association of low immune-evasion capacity (defined as tumors with no immune editing potential, no HLA loss and no antigen processing machinery [APM] defects) and high number of neoantigens with increased disease-free survival [14].

Moreover, the release of checkpoint blockade by nivolumab may result in a series of dynamic changes in the composition of the tumor microenvironment [15] which override the current prediction (false negatives), but this was not taken into account as we were limited to the use of archival material prior to ICI therapy. Notably, a significant correlation between PD-L1 expression and TML was determined, which may contradict accumulating evidence from clinical trials [16]. This may be related to the limited number of patients, but may also result from the use of continuous covariates rather than stratified data in clinical trials where true correlations may easily be overlooked. Based on our findings, it could not be established that TML and PD-L1 serve as an independent biomarker for clinical outcome.

An interesting finding of this study is the added value of expression of HLA class I molecules on cancer cells, which is known to be crucial for the recognition of tumor cells by CD8 + TCs. In our opinion, the actual detection of HLA class I expression is more valuable as a future biomarker for ICIs than genetic, epigenetic, transcriptional, post-transcriptional or post-translational aberrations, such as loss of heterozygosity in *HLA* or *B2M* mutations, since protein expression is the ultimate outcome of all those changes. For instance, genetic studies have revealed that *NLRC5*, an HLA class I transactivator, is an important target for cancer immune evasion. The expression of *NLRC5* correlated with that of HLA class I and negatively correlated with OS in stage III NSCLC [17]. We focused primarily on the expression of HLA class I and did not determine selective APM defects, while this could also affect the recognition of tumor antigens by the immune system. For HLA peptides to be presented to CD8 + T cells, peptides must be processed by proteolysis, trimmed by enzymes to fit into the groove of HLA molecules, and transported intracellularly by peptide transporters, endoplasmic reticulum chaperones and the Golgi apparatus. The antigen presenting pathway often is altered in cancer, including lung cancer [18, 19]. Further studies should be directed at investigating the impact of the APM defects on response to ICIs. Last but not least, due to the relatively low patient numbers, we decided to take complete loss defined as a low score (0–3) of both HLA-A and HLA-B/C, but not partial HLA class I loss into consideration. However, a subgroup analysis showed that patients with complete loss have impaired OS and PFS compared to patients with no loss of HLA class I. The PFS of patients with partial loss was comparable to that of patients with no loss of HLA class I.

Taken together, the findings support the hypothesis that a rational combination of biomarkers—based on the biological requirements for the ICIs to work—may contribute to a more adequate response prediction of ICI treatment in NSCLC. Consequently, the refinement of this proposed set of biomarkers and validation in a greater set of patients is warranted.

### Electronic supplementary material

Below is the link to the electronic supplementary material.

Supplementary file1 (PDF 877 kb)

## Abbreviations

APM: Antigen processing machinery
CR: Complete response
FFPE: Formalin-fixed, paraffin-embedded
ICI: Immune checkpoint inhibitor
NGS: Next-genome sequencing
NSCLC: Non-small-cell lung cancer
PD: Progressive disease
PR: Partial response
SD: Stable disease
TML: Tumor mutational load
TPS: Tumor proportion score
