# Supplementary material for: Tumor mutational load, CD8+ T cells, expression of PD-L1 and HLA class I to guide immunotherapy decisions in NSCLC patients
Source: Cancer Immunol Immunother. 2020 Feb 12;69(5):771–7. doi: 10.1007/s00262-020-02506-x (PMC7183487; doi:10.1007/s00262-020-02506-x)
Supplement: Supplementary file 1 — Supplementary file1 (PDF 877 kb) [file 262_2020_2506_MOESM1_ESM.pdf]

supplementary Table 1

	Response		No response		
	**Number (%)**		**Number (%)**		**p-value**
TML					0.043
Low (<11 mut/Mb)	5	(45.5%)	12	(85.7%)	
High (>11 mut/Mb)	6	(54.5%)	2	(14.3%)	
HLA-A					0.4
Low	4	(40.0%)	10	(52.6%)	
High	6	(60.0%)	9	(47.4%)	
HLA-B/C					0.615
Low	4	(40.0%)	8	(42.1%)	
High	6	(60.0%)	11	(57.9%)	
Total $CD8^+$					0.005
Low	2	(20.0%)	14	(77.8%)	
High	8	(80.0%)	4	(22.2%)	
PD-L1 (TPS)					0.492
Neg (0%)	5	(45.5%)	6	(37.5%)	
Pos (>1%)	6	(54.5%)	10	(62.5%)	
PD-L1 (TPS)					0.279
Low (<50%)	7	(63.6%)	13	(81.3%)	
High (>50%)	4	(36.4%)	3	(18.8%)	

Best overall response analysis.

Patient grouping by response to anti-PD1 ICI therapy, comparing TML low vs. high, HLA-A low vs. high, HLA-B/C low vs. high, total $CD8^+$ low vs. high, PD-L1 negative (neg) vs. positive (pos), PD-L1 low (<50%) vs. high (>50%). P-values (1-tailed) by Fisher's Exact test.

supplementary Table 2

	**Response**		**Non-response**		
	Mean (SE)		Mean (SE)		p-value
TML	10.2	(1.0)	5.2	(0.9)	0.001
HLA-A	5.2	(0.9)	3.2	(0.8)	0.098
HLA-B/C	4.8	(0.8)	3.9	(0.7)	0.407
Total $CD8^+$	58.5	(9.2)	18.5	(8.4)	0.004
PD-L1 (TPS)	23.0	(10.0)	16.3	(9.1)	0.629

Test of between-subjects effects.

Tests of Between-Subjects Effects (n=22) of response comparing individual predictive absolute values of: TML (mut/Mb), HLA-A and HLA-B/C (Ruiter scoring), total $CD8^+$ (cells/mm^2), PD-L1 (TPS; %). P-values (1-tailed) by Multivariate testing, excluding patients with missing values.

supplementary Table 3

		PD-L1	Total CD8+	TML	HLA-A	HLA-B/C
**PD-L1 (TPS; %)**	Correlation Coefficient		0.022	,440*	-0.184	-0.227
	Sig. (2-tailed)		0.916	0.035	0.240	0.147
	Number		25	23	26	26
**Total CD8+ (cells/mm2)**	Correlation Coefficient	0.022		0.067	0.163	0.086
	Sig. (2-tailed)	0.916		0.754	0.249	0.546
	Number	25		24	28	28
**TML (mut/Mb)**	Correlation Coefficient	,440*	0.067		0.064	-0.035
	Sig. (2-tailed)	0.035	0.754		0.682	0.820
	Number	23	24		24	24
**HLA-A (Ruiter scoring)**	Correlation Coefficient	-0.184	0.163	0.064		,623**
	Sig. (2-tailed)	0.240	0.249	0.682		0.000
	Number	26	28	24		29
**HLA-B/C (Ruiter scoring)**	Correlation Coefficient	-0.227	0.086	-0.035	,623**	
	Sig. (2-tailed)	0.147	0.546	0.820	0.000	
	Number	26	28	24	29	

Correlation matrix.

Correlation matrix of PD-L1 tumor proportional score (TPS; %), total CD8+ T cell infiltration (cells/mm^2), TML (mutations/Mb), HLA-A and HLA-B/C (Ruiter Score). Parametric correlations were performed by Pearson correlation coefficient, non-parametric correlations by Kendall's Tau. TML was significantly correlated with PD-L1 (r^2=0.440, p=0.035), HLA-A was significantly correlated with HLA-B/C (r^2=0.623, p=0.000001).

supplementary Figure 1

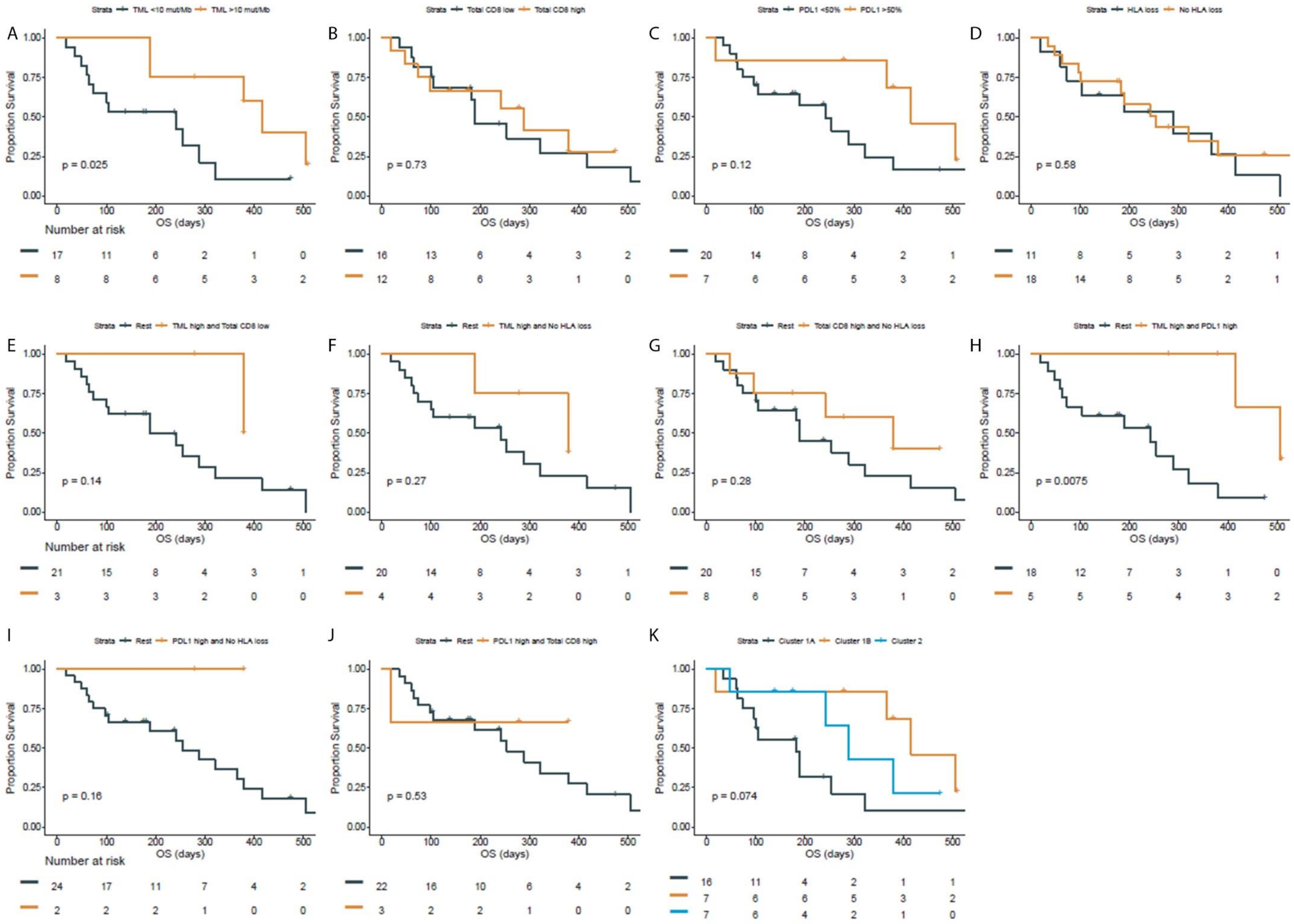

OS analysis.

Kaplan-Meier plots showing the overall survival by **a** TML high (>11 mut/Mb) vs. low (<11 mut/Mb), **b** CD8^{+} T cell infiltration high vs. low, **c** PD-L1 high (>50%) vs low, **d** classical HLA (-A and –B/C) loss vs. rest, **e** TML high and total CD8^{+} high vs. rest, **f** TML high and no loss of classical HLA vs. rest, **g** total CD8^{+} high and no loss of classical HLA vs. rest, **h** TML high and PD-L1 high vs. rest, **i** PD-L1 high and no loss of classical HLA vs. rest, **j** PD-L1 high and CD8^{+} high vs. rest, **k** cluster (cluster 1A, 1B and 2).

supplementary Figure 2

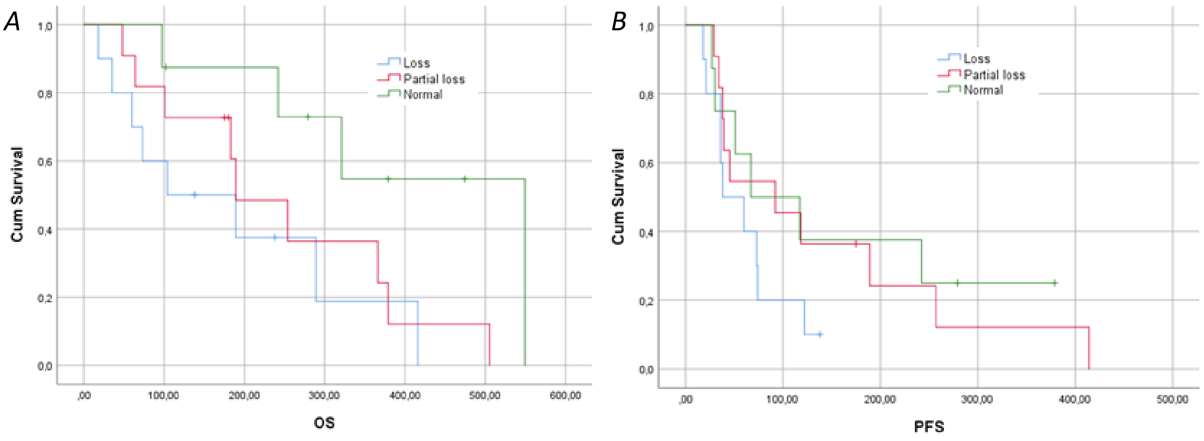

Kaplan-Meier curves based on HLA class I expression.

Kaplan-Meier curves showing the ***a*** *OS en* ***b*** *PFS of patients based on the HLA class I expression: complete loss vs. partial loss vs. no loss of HLA class I. Here, complete loss was defined as 0-2, partial loss as 3-6, and no loss as 7-8. This resulted in a separate score for HLA-A and HLA-B/C. These scores were combined into a single score for HLA class I expression. Statistical tests were non-significant for OS (log rank p=0.055) and PFS (log-rank p=0.298).*
